# The Association of HLA Class 1 and Class 2 Antigens with Multiple Myeloma in Iranian Patients

**DOI:** 10.4274/tjh.2013.0098

**Published:** 2014-12-05

**Authors:** Arezou Sayad, Mohammad Taghi Akbari, Mahshid Mehdizadeh, Elham Roshandel, Soheila Abedinpour, Abbas Hajifathali

**Affiliations:** 1 Shahid Beheshti University of Medical Sciences, Department of Medical Genetics, Tehran, Iran; 2 Tarbiat Modares University Faculty of Medical Science, Department of Medical Genetics, Tehran, Iran; 3 Shahid Beheshti University of Medical Sciences, Taleghani Bone Marrow Transplantation Center, Tehran, Iran; 4 Shahid Beheshti University of Medical Sciences, Pediatric Congenital Hematologic Disorders Research Center, Tehran, Iran

**Keywords:** Multiple myeloma, HLA-A, HLA-B, HLA-DRB1, Genetic susceptibility

## Abstract

**Objective:** Multiple myeloma (MM) is a B-cell malignancy characterized by the clonal proliferation of malignant plasma cells. According to results of some studies, it has been suggested that the HLA class 1 and 2 genes have susceptibility effects on MM. Studies of different populations have reported different HLA class 1 and 2 alleles that affect MM. In this study, we assessed the association of HLA class 1 and class 2 antigens with MM in Iranian patients.

**Materials and Methods:** We performed a case-control genotyping study with 105 Iranian MM patients that were selected from the bone marrow transplantation department of Taleghani Hospital and 150 controls using single specific primer-polymerase chain reaction with the HLA-Ready Gene ABDR Kit.

**Results:** Our results demonstrated that 21% of patients versus 12% of controls and 11% of patients versus 3% of controls carried HLA-A*03 and HLA-B*18, respectively. The MM patients had a significant increase in the frequency of HLA-A*03 and HLA-B*18 alleles in comparison to control subjects (p=0.039, OR=2.057 and p=0.013, OR=3.567, respectively).

**Conclusion:** Our findings suggested that the HLA-A*03 and HLA-B*18 alleles have significant susceptibility effects on MM in the Iranian population. However, compared to other populations, the above-mentioned alleles had different statuses. Since there are not many studies evaluating and calculating this association among ethnic groups, further studies among other populations are needed to explain the exact association of the HLA genes with MM.

## Abstract (tr)

**Amaç:** Multiple myeloma (MM), malign plazma hürelerinin klonal çoğalması ile karakterize bir B hücre neoplazisidir. Çeşitli çalışmaların sonuçlarına göre, bazı sınıf 1 ve 2 HLA genlerinin hastalığa yatkınlık sağladığına dair görüşler ortaya atılmıştır. Farklı popülasyonlarda yapılan çalışmalarda, farklı HLA sınıf 1 ve 2 allellerinin MM üzerine etkisi olduğu bildirilmiştir. Bu çalışmada, İranlı MM hastalarında HLA sınıf 1 ve sınıf 2 antijenlerinin birlikteliğini değerlendirdik.

**Gereç ve Yöntemler:** HLA-Ready Gene ABDR kitleriyle tekli spesifik primer polimeraz zincir reaksiyonu yönteminin kullanıldığı bu olgu-kontrol genetiplendirme çalışmasında, hasta grubuna Taleghani Hastanesi kemik iliği nakli bölümünden seçilen 105 İranlı MM hastası ve 150 de kontrol olgusu dahil edilmiştir.

**Bulgular:** Çalışma sonucunda, HLA-A*03 hasta grubunda %21 ve kontrol grubunda %12 bulunurken, HLA-B*18 ise hasta grubunda %11 ve kontrol grubunda %3 olarak saptanmıştır. MM hastalarının HLA-A*03 ve HLA-B*18 allele sahip olma oranı kontrol olgularıyla karşılaştırıldığında istatistiki olarak anlamlı olacak şekilde yüksek bulunmuştur (p=0,039, OR=2,057 ve p=0,013, OR=3,567, sırasıyla).

**Sonuç:** Bizim bulgularımız, İran toplumunda HLA-A*03 ve HLA-B*18 allel varlığının istatistiki olarak anlamlı olacak şekilde MM’ye yatkınlık yarattığını ortaya koymaktadır. Bununla birlikte, diğer toplumlara bakıldığında adı geçen allellerin aynı sonucu doğurmadıkları görülmektedir. Farklı etnik gruplar arasındaki bu birlikteliği değerlendiren fazla sayıda çalışma olmadığı için, gelecek dönemlerde MM’li hastalarda HLA genlerinin birlikteliğinin sonuçlarını izah edebilecek daha ayrıntılı çalışmalara gereksinim vardır.

## INTRODUCTION

Multiple myeloma (MM) is a B-cell malignancy characterized by the clonal proliferation of malignant plasma cells, and evidence indicates that the bone marrow microenvironments of tumor cells have a crucial role in myeloma pathogenesis [1]. Neurological and impaired hemopoiesis symptoms, bone complications, renal failure, and infection are some of the heterogeneous clinical features [2]. MM is the second most prevalent blood cancer after non-Hodgkin lymphoma [3]. MM represents approximately 1% of all cancers, 2% of all cancer deaths, and 10% of hematological malignancies. The prevalence of MM varies among different populations. Blacks have a 2-fold higher incidence than whites, while the Japanese, Chinese, and South Koreans have the lowest incidence [4,5]. Although the exact etiology of MM is unknown, the genetic factor has an important effect on it. Simply, MM is a condition characterized by the unlimited proliferation of plasma cells. Since the HLA genes are associated with a variety of immunologic diseases, they may be involved as a crucial factor in MM [6]. In 1970 for the first time, after recognition of HLA class 1 and prior to identification of HLA class 2, the susceptibility effects of HLA genes on MM were studied [7,8,9,10,11,12]. Not many studies have taken sufficient account of the effect of HLA genes on the susceptibility to MM in different populations. These studies reported different susceptible or protective alleles of HLA genes in association with MM. Some studies indicated no significant association between HLA-A and -B genes and MM, while other studies demonstrated that HLA-A3, -B18, -Bw65, and -DRw14 had associations with MM [13,14,15]. In the present study, for the first time, the associations of HLA class 1 and 2 genes with MM in Iranian patients were investigated.

## MATERIALS AND METHODS

**Patients and Controls**

One hundred and five Iranian patients were selected from the bone marrow transplantation department of Taleghani Hospital. The diagnosis of MM was made by an oncologist. Additionally, one hundred and twenty ethnically, age-, and sex-matched healthy individuals without personal or familial history of cancer or autoimmune disorders were included as controls. The subjects gave informed consent to participate.

**DNA Extraction and HLA Genotyping**

Genomic DNA from venous peripheral blood samples was extracted by the salting-out method [16]. HLA typing was performed at the Tehran Medical Genetics Laboratory. HLA-A, -B, and -DRB1 genotyping was carried out based on low-resolution single specific primer-polymerase chain reaction (SSP-PCR) HLA typing with the HLA-Ready Gene ABDR Kit (Inno-Train Diagnostik GmbH, Germany) according to the manufacturer’s recommendation. The PCR products were run on 2% agarose gel.

**Statistical Analysis**

By using chi-square and Fisher exact tests, comparisons between HLA-A, -B, and -DRB1 alleles of MM patients and the controls were performed. SPSS 18.0 for Windows was used for analysis and p<0.05 was considered to be statistically significant. The p-value was corrected with Bonferroni correction. In statistics, the Bonferroni correction is a method used to counteract the problem of multiple comparisons. Odds ratio (OR) and 95% confidence interval (CI) were determined.

## RESULTS

Distributions of sex and age of MM patients and the controls are shown in Table 1. The allele frequencies of HLA-A, -B, and -DRB1 in MM patients and controls are demonstrated in Table 2. As outlined in Table 2, no significant associations between HLA-DRB1 and MM were observed. Low-resolution HLA typing revealed that 21% of patients versus 12% of controls and 11% of patients versus 3% of controls carried HLA-A*03 and HLA-B*18, respectively. The MM group had a significant increase in the frequency of HLA-A*03 and HLA-B*18 alleles in comparison to control subjects (p=0.039, OR=2.057 and p=0.013, OR=3.567, respectively) (Table 2).

## DISCUSSION

After description of a serological technique for HLA typing, studies on the association of the HLA genes with susceptibility to or protection against different disease were begun. In our research, for the first time, we investigated the association of HLA class 1 and 2 genes with MM disease in Iranian patients. In our study, the HLA-A*03 and HLA-B*18 alleles had higher frequencies in MM patients than in control individuals and had significantly positive associations with MM. Therefore, the HLA-A*03 and HLA-B*18 alleles have a susceptibility effect in Iranian MM patients (p=0.039 and OR=2.057, p=0.013 and OR=3.567, respectively).

Consistent with our results, in 2002, a study on 68 MM patients in southern Africa reported that the HLA-B*18 allele had an association (p<0.005, OR=6.3) and HLA-DRB1 had no association with MM significantly. In contrast to our results, however, that study found that there was no statistically significant association between HLA-B and MM [14]. Patel et al. demonstrated no significant association between antigens at either the A or the B locus in MM patients compared to controls [15]. Additionally, in a study on black and white men in 1992, it was shown that black MM patients had significantly higher HLA-Bw65 and HLA-DRw14 allele frequencies than black controls, while white MM patients had a higher A3 allele frequency than white controls [13]. A study on German, Dutch, American, and English subjects showed a weak association between HLA-B5 and MM [8,9,10,17]. No significant HLA-A and -B allele associations were demonstrated in French and Swiss MM patients, although the MM patients from another area of France showed a negative association between HLA-Aw32 and MM [6,7,12]. Ludwig and Mayr, in 1982, reported that comparisons between all available studies showed a significantly increased frequency of HLA-B5 [18].

HLA allele associations in our population demonstrated some differences from previous published studies on MM. Variations among ethnic groups may support these differences. Not many studies have taken sufficient account of the association between HLA and MM. Moreover, there are not many studies that have evaluated and calculated this association among ethnic groups. It is suggested that other populations be studied to find the correlation of HLA and MM to find out the exact association.

## CONCLUSION

The HLA-A*03 and HLA-B*18 alleles have significant susceptibility effects on MM in the Iranian population. However, compared to other populations, the above-mentioned alleles had different statuses. There are other reports that show both consistency and inconsistency with our results. Ethnic variations among different populations in part explain these controversies. Further studies with large sample sizes or family studies are needed to confirm the exact associations of HLA genes with MM.

**Conflict of Interest Statement**

The authors of this paper have no conflicts of interest, including specific financial interests, relationships, and/or affiliations relevant to the subject matter or materials included.

## Figures and Tables

**Table 1:**
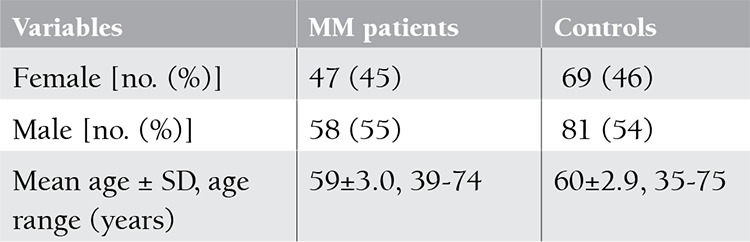
Distributions of sex and age of multiple myelom (MM) patient and control groups.

**Table 2:**
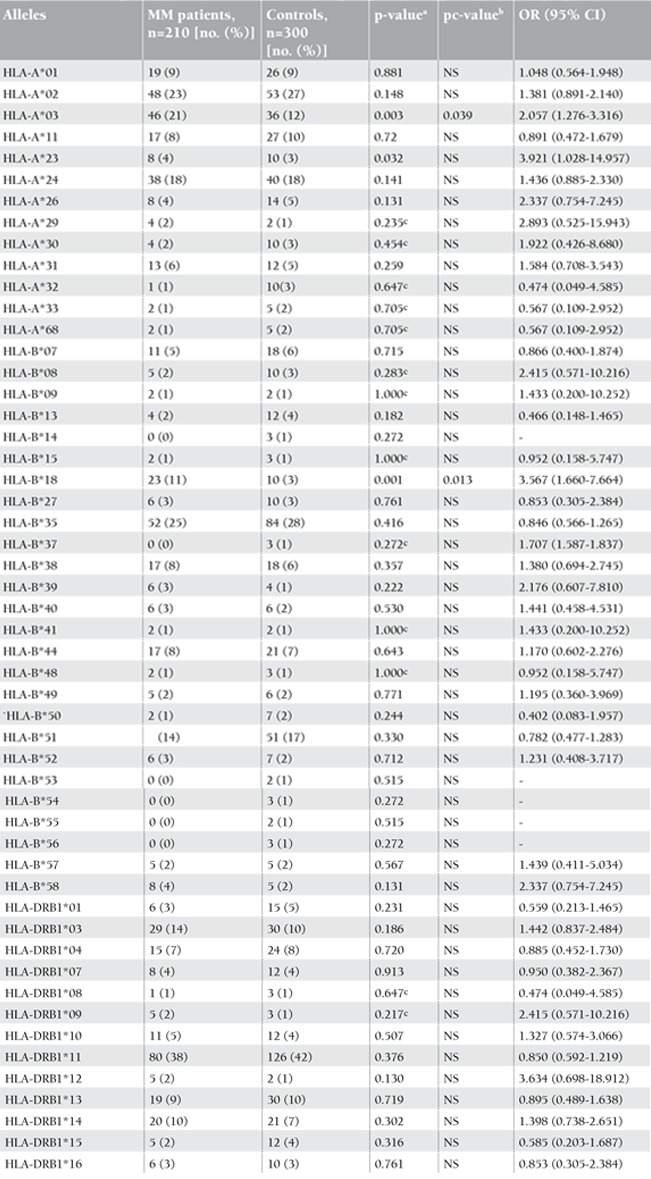
The allele frequencies of HLA-A, -B, and -DRB1 in multiple myelom (MM) patient and control groups.
